# Scalable and customizable parallel flow-through reactors to quantify biological processes related to contaminant attenuation by photosynthetic wetland microbial mats

**DOI:** 10.1016/j.mex.2023.102074

**Published:** 2023-02-14

**Authors:** Gary Vanzin, Henry Peel, Weishi Wang, Lily Bosworth, Zhaoxun Yang, Michael A.P. Vega, Colin Root, Adam Brady, Giuliana Romero Mariscal, Armando Arenazas Rodríguez, Juana Ticona, Lino Morales Paredes, Jonathan O. Sharp

**Affiliations:** a Department of Civil and Environmental Engineering, Colorado School of Mines, CO 80401, United States; b Centro de Minería Sostenible, Universidad Nacional de San Agustín de Arequipa, Arequipa 040000, Peru

**Keywords:** Engineered wetlands, Field-scale, Laboratory-scale, Bioremediation, Algae, Nature-based treatment, Scalable and customizable parallel flow-through reactors to quantify biological processes related to contaminant attenuation by photosynthetic wetland microbial mats

## Abstract

Shallow, unit process open water wetlands harbor a benthic microbial mat capable of removing nutrients, pathogens, and pharmaceuticals at rates that rival or exceed those of more traditional systems. A deeper understanding of the treatment capabilities of this non-vegetated, nature-based system is currently hampered by experimentation limited to demonstration-scale field systems and static lab-based microcosms that integrate field-derived materials. This limits fundamental mechanistic knowledge, extrapolation to contaminants and concentrations not present at current field sites, operational optimization, and integration into holistic water treatment trains. Hence, we have developed stable, scalable, and tunable laboratory reactor analogs that offer the capability to manipulate variables such as influent rates, aqueous geochemistry, light duration, and light intensity gradations within a controlled laboratory environment. The design is composed of an experimentally adaptable set of parallel flow-through reactors and controls that can contain field-harvested photosynthetic microbial mats (“biomat”) and could be adapted for analogous photosynthetically active sediments or microbial mats. The reactor system is contained within a framed laboratory cart that integrates programable LED photosynthetic spectrum lights. Peristaltic pumps are used to introduce specified growth media, environmentally derived, or synthetic waters at a constant rate, while a gravity-fed drain on the opposite end allows steady-state or temporally variable effluent to be monitored, collected, and analyzed. The design allows for dynamic customization based on experimental needs without confounding environmental pressures and can be easily adapted to study analogous aquatic, photosynthetically driven systems, particularly where biological processes are contained within benthos. The diel cycles of pH and dissolved oxygen (DO) are used as geochemical benchmarks for the interplay of photosynthetic and heterotrophic respiration and likeness to field systems. Unlike static microcosms, this flow-through system remains viable (based on pH and DO fluctuations) and has at present been maintained for more than a year with original field-based materials.

• Lab-scale flow-through reactors enable controlled and accessible exploration of shallow, open water constructed wetland function and applications.
• The footprint and operating parameters minimize resources and hazardous waste while allowing for hypothesis-driven experiments.
• A parallel negative control reactor quantifies and minimizes experimental artifacts.

## Graphical abstract

**Figure:**
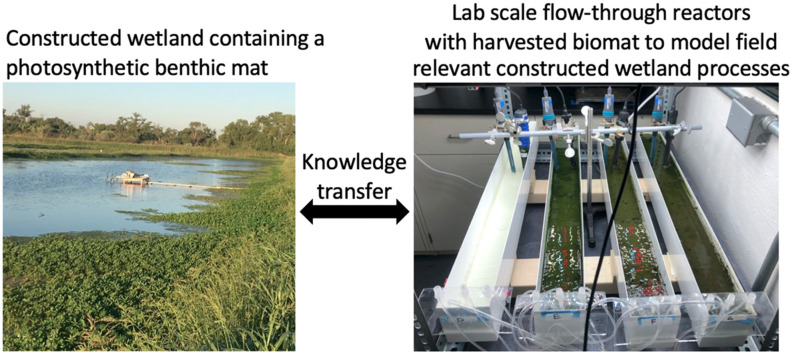

Specifications table

**Table:** 

Subject area:	Environmental Science

More specific subject area:	Environmental Engineering
Name of your method:	Scalable and customizable parallel flow-through reactors to quantify biological processes related to contaminant attenuation by photosynthetic wetland microbial mats
Name and reference of original method:	A. Brady, M. Vega, K. Riddle, H. Peel, E. Lundeen, J. Siegmund, J. Sharp, Biomat Resilience to Desiccation and Flooding Within a Shallow, Unit Process Open Water Engineered Wetland, Water. 13 (2021) 815. 10.3390/w13060815
Resource availability	Appendix A: Material Purchasing Links Supplemental information: Reactor volume and flow calculations

## Method details

### Background

Field-scale unit process open water wetlands (UPOWs) provide a novel alternative to vegetated treatment wetlands. They have been shown to effectively attenuate trace organic pharmaceuticals, nutrients, and pathogens that remain after municipal wastewater treatment, as well as pesticides introduced in runoff [1], [2], [3], [4], [5], [6], [7], [8]. UPOWs have been sustainably integrated into urban infrastructure, where habitat and aesthetic gains are achieved without the external energy demands of more conventional engineered treatment solutions for these constituents. A primary characteristic of UPOWs are a benthic liner that limits emergent macrophyte growth, and a shallow (< 30 cm) water column. The photosynthetic biomat that colonizes this liner is a complex assemblage of diatoms and bacteria [9] that reproducibly colonize within months of initial operation without the need for external bioaugmentation [10]. Demonstration field-scale systems have remained operationally and microbially stable over a period of years and demonstrate reestablishment and functional resilience after operational disturbances [10,11]. As a complement to field-scale operation, further laboratory experimentation using field-derived materials that capture flow and diel dynamics enables greater fundamental mechanistic knowledge, extrapolation to contaminants and concentrations not present at current field sites, operational optimization, baseline reproducibility between laboratory groups, capability to scale volumetrically, and integration into holistic water treatment trains.

**Fig. 1:**
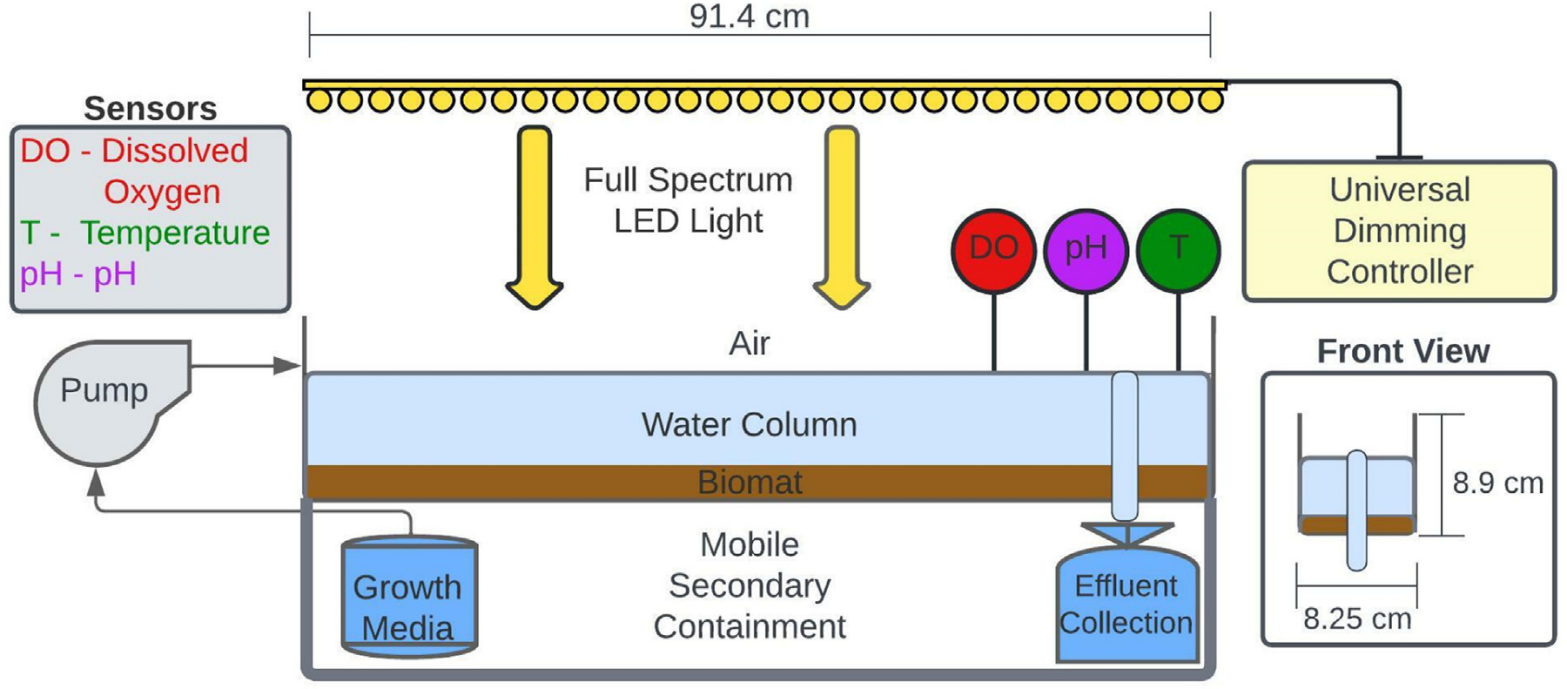
Laboratory scale biological reactor with programmable LED lights (spectrum: 3000 K, 5000 K, red 660 nm and IR730 nm), secondary containment and instrumented with appropriate aqueous probes (pH, temperature, and dissolved oxygen).

The design presented here advances continuous flow reactor microcosms briefly described by Brady et al. [11], which consisted of rectangular Pyrex trays (∼20 × 15 × 5 cm) seeded with fresh and rehydrated biomat from the Prado Constructed Wetlands (Orange County, California). Other iterations of modeled wetlands included batch reactors, but these lack the continuous flow and constant reintroduction of nutrients needed for steady-state microbial viability, limiting experimental applications to a timeframe of days without periodic augmentation. Although other flow through lab wetland model systems have been described [12], their traditional vegetated design precludes studying photolysis and photoinactivation due to plant shading, while dealing with plant-driven short-circuiting that may not replicate field conditions. This flow-through reactor design is better suited to mimic UPOW field conditions because of the continuous water flux, gravity-driven effluent removal, and the longevity of the system. Typical use cases include evaluating nutrient, pharmaceutical, and / or metal transformation under various operational conditions [13]. The final design (Fig. 1) is leakproof and self-contained, which can be critical if the model systems are challenged with hazardous contaminants. LED grow lights can be controlled to modulate the wavelengths, intensity, and duration of light. Although the current design uses ‘full spectrum’ LEDs, UV-free LEDs help deconvolute biotic and abiotic processes by minimizing the photolysis of organic contaminants while still supporting photosynthesis [9]. In addition, the final design uses plastic, coated metals and stainless steel parts, which minimizes experimental artifacts such as unintentionally introduced dissolved metals. Substitute materials, such as plexiglass flow-through reactors instead of metal ones (Fig. 10), should maintain the integrity of the method and conform to the experimental goals. The scalable laboratory flow-through analog offers the ability to manipulate variables such as influent rates, aqueous geochemistry, light duration, and light intensity gradations within a controlled laboratory environment in order to rapidly and accurately assess field-scale potential without jeopardizing environmental waters. Although our method is tailored to understanding reactions occurring in UPOW wetlands, it could also be applied to other flowing, benthic ecosystems such as lakes, streams, and estuaries.

### Design and construction

All required materials are found in Table 1 with purchasing links available in Appendix A.

**Table 1:** Flow reactor materials list – citations refer to purchasing links included in the supplementary information.

**Description**	**Quantity**
Flow-through reactors–32″D x 3.25″W x 3.5″H Metal Planter ^1^	1
Grow Lights Spectrum: 3000 K, 5000 K, red 660 nm, and IR730nm ^2^	1
Dimming Controller ^3^	1
Utility cart ^4^	1
1/4″ Stainless Bulkhead Union ^5^	1
1/4″ Stainless Steel Ferrule Set ^6^	2
Stainless Steel Tubing ^7^	Varies
2-Stop Tubing (Order # SC0016) ^8^	2
Masterflex Transfer Tubing: 1/4″ ID^9^	1
Assorted Tube Connector Kit ^10^	1
Ismatec IP or IP-N Peristaltic Pump ^11^	1
½ in. Rubber Gasket ^12^	1
Plumber's Putty ^13^	1
Furniture Levels (Optional)^14^	4–6
Rubber gasket ½ in	1

### Flow-through reactor drain installation

**Fig. 2:**
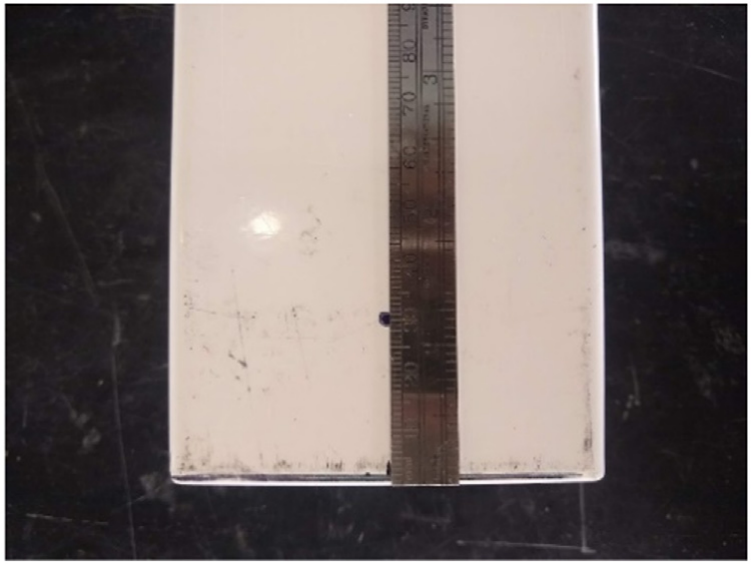
Measurements to demark a point on the bottom of the planter for a drilled effluent port.
(2) Drill a 1/2″ (1.25 cm) hole with a drill press or powerful hand drill. Use a center punch to indent before drilling. If using a hand drill, it may be beneficial to drill a smaller hole first. Use a deburring tool to remove sharp edges.
(3) Install a rubber gasket and plumbers putty on a Swagelok 1/4″ (0.635 cm) stainless steel bulkhead unit. Rubber gaskets ½ in. (1.25 cm) can be purchased from most hardware stores, the one in Fig. 3 was taken from the seal on a 2 mL plastic cryotube. Roll the gasket onto the Swagelok and attach the plumber's putty underneath roughly the same size as the gasket, as shown in Fig. 3.

(1) Mark the bottom of the planter to drill in the center and 3 cm from the end (Fig. 2)*.*

**Fig. 3:**
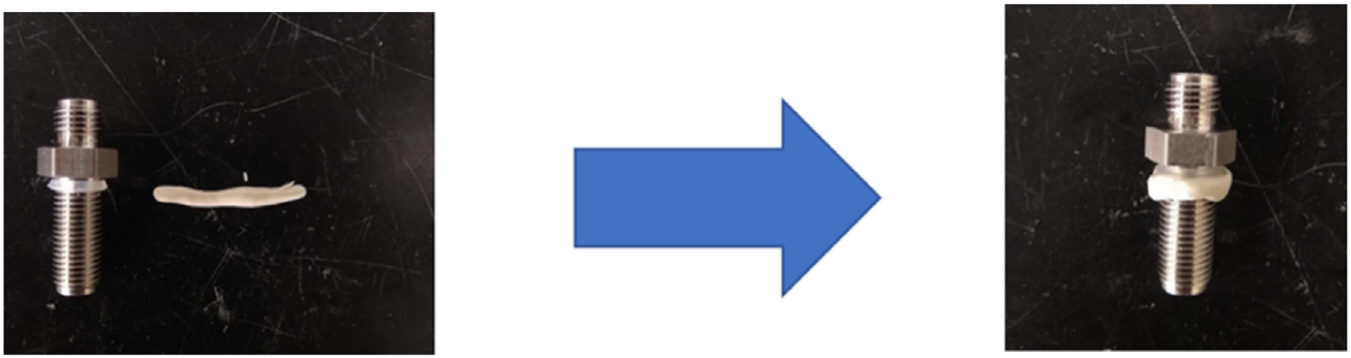
The addition of a rubber gasket and plumber's putty to the Swagelok fitting.
(4) Slide the Swagelok 1/4″ (0.635 cm) Stainless Bulkhead Union into the hole with the gasket and putty on the interior bottom of the planter. Screw the nut that came with the Swagelok undeheath until tight. Remove the excess plumber's putty that was pushed out of the sides.
(5) If you want the height of the water column to be higher than that provided by the Swagelok fitting, use a hack saw and vice or a vertical band saw to cut the steel tubing (Fig. 4) to size. Use a file to level out and smooth the cut.

**Fig. 4:**
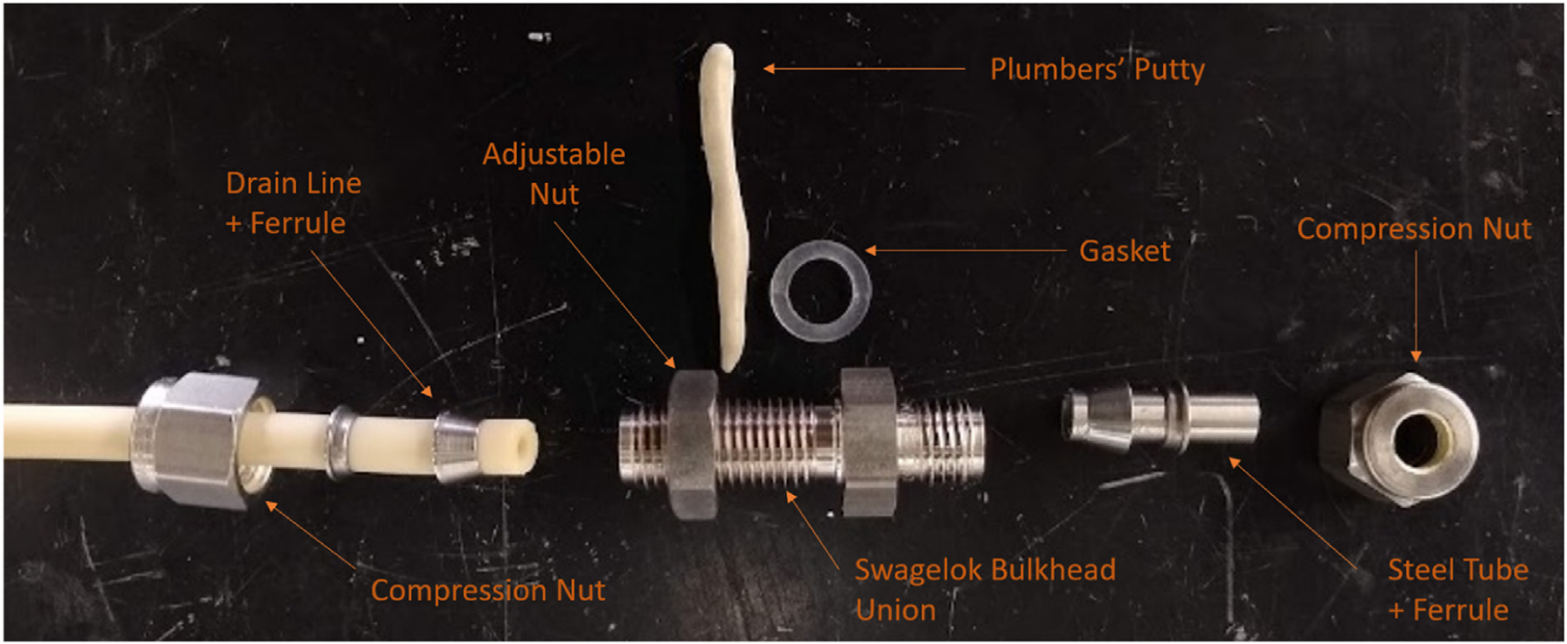
Decoupled effluent port plumbing.
(6) Use the ferrule set and the compression nuts that came with the Swagelok to attach the stainless steel tube as shown in Figs. 4 and 5. Fig. 6 shows the drain attached to the planter.

**Fig. 5:**
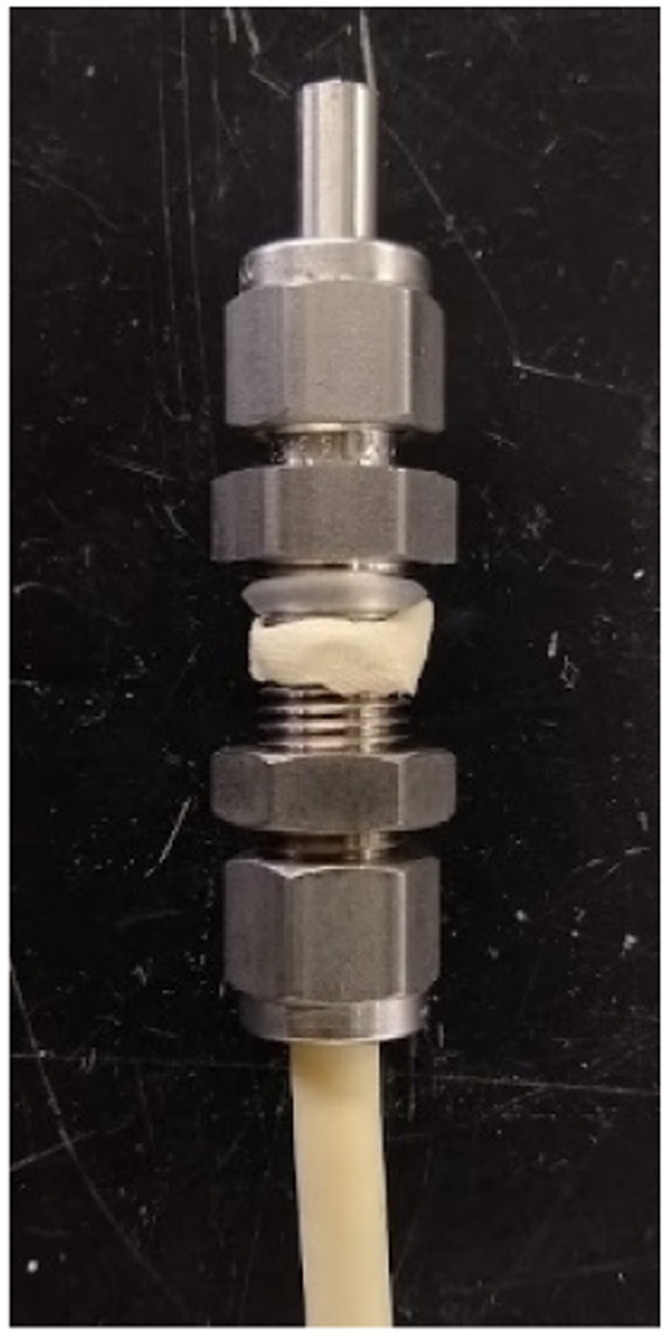
Assembled gravity-driven effluent port. Note that the planter box is located below the plumber's putty and the nut is on the opposite surface (bottom) of the planter box.

**Fig. 6:**
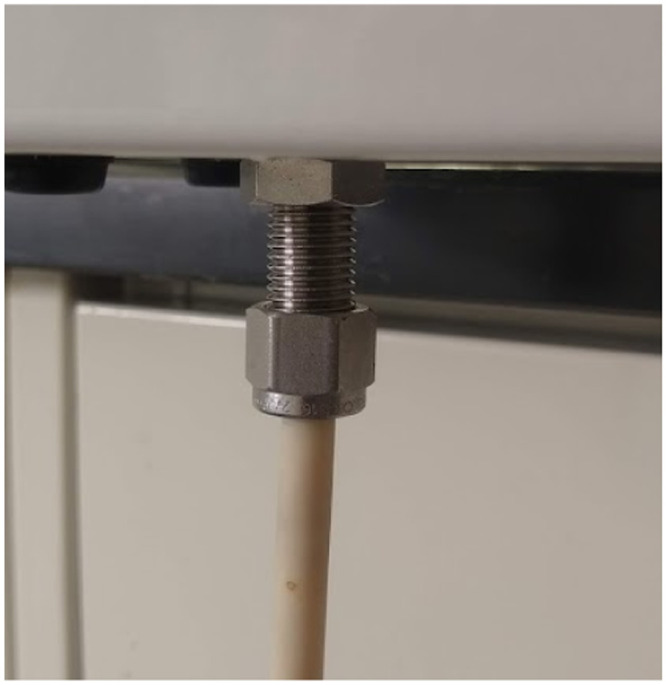
The downstream portion of the gravity-driven effluent port attached to the lower surface of the planter.
(7) To check for leaks, fill the container with water and monitor the planter for at least an hour.
(8) Attach the influent lines to the flow-through reactor. We used plexiglass shaped with insert holders from a previous experiment (Fig. 7), drilled with small holes at the inlet to hold the tubes. Use tape to hold the tubing in place, ideally above the water column, to prevent contamination of the inlet tubing.

**Fig. 7:**
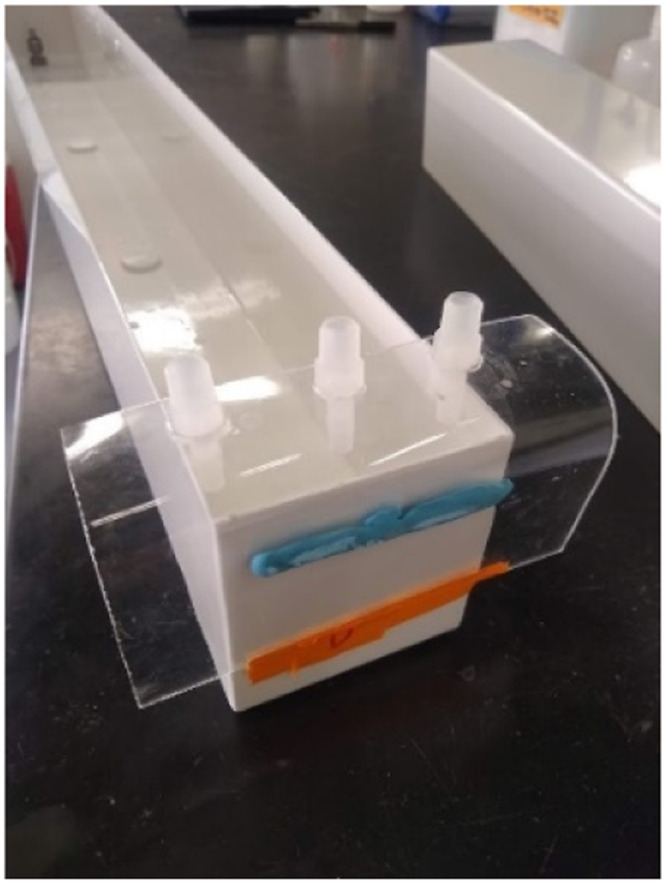
Bent plexiglass tube holder for the introduction of multiple influent lines.

### Mobile cart to contain reactor system

Placing the reactors on a mobile cart is optional, but recommended for easy transport of the system. Cart trays also act as built-in secondary containment.

(1) Assemble the mobile cart according to the manufacturer's instructions.
(2) Drill a ¾ in. (1.9 cm) hole for the drain on the top shelf of the cart at the end away from the handles. The holes are evenly spaced according to the number of flow-through reactors used, so they are centered on the cart. We have successfully fit four on a 36 in. (91.44 cm) by 24 in. (60.96 cm) utility cart. If level reactors are a concern, a frame can be placed underneath the planters with furniture leveling pads.

### Lighting

We recommend using a frame that fits the cart to hold the grow lights. We used zinc-plated steel angle posts and added a plexiglass shelf above the light for the storage of probes, meters and cords. Wood, T-slot rails, or plastic are all fine options for building the frame. It is fine to use what is on hand, inexpensive, or easily accessible. The outer dimensions for a tight-fitting frame are 0.6 m (24″) width, 0.9 m (36″) length, and 0.6 m (24″) height. The lights are 0.5 m (18″) above the surface of the flow-through reactors water. This provides a maximum Photosynthetic Photon Flux Density (PPFD) of 706 mmol/s/m^2^ in the center and 353 mmol/s/m^2^ at the outer corners of a 4 reactor set-up. While this is less PPFD than at the Prado Constructed Wetlands in August (e.g. 1058 mmol/s/m^2^ at 10AM, 1449 mmol/s/m^2^ at 1:30PM, and 1090 mmol/s/m^2^ at 3:00PM), the flow-through reactors require less light to achieve similar diel cycling as the field wetland due to the shallow and clear water column created under laboratory conditions (Fig. 11). An improvement in the design could be the use of multiple light banks for more uniform coverage. The dimmer control allows for gradual increase and decrease in light intensity for defined light and dark time periods, to mimic sunrise and sunset. Note that if a dimming controller is used, the light fixture needs to have 0–10 V dimmable LEDs and the appropriate driver. Although we typically use 12 h on and 12 h off with one-hour light ramps, this is a tunable variable that can be adjusted to better match desired conditions and time of year. To assemble the light fixture:

(1) Ensure safety during electrical work. If unfamiliar with the tasks hire a professional, such as an electrician.
(2) Install the full-spectrum LED growing lights according to the manufacturer's instructions.
(3) Use wire cutters to cut the cord just below the dimmer switch. Strip the wires and attach them to the dimming controller.
(4) Attach the light to the frame using the hangers that come with the grow light.
(5) Optional: Attach the LED dimmer controller to the frame or cart using zip ties, utility cord, nuts and bolts, or whatever is readily available and provides a strong attachment in a convenient location (Fig. 9).

### Pump and inlet tubing

**Fig. 8:**
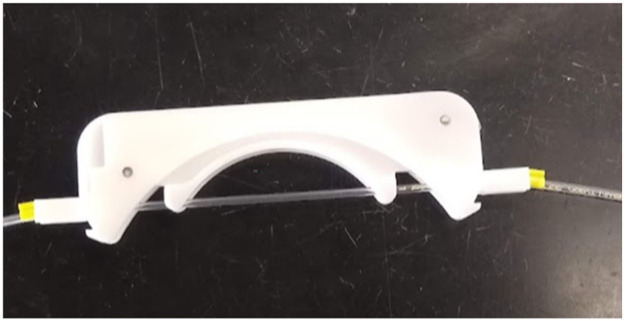
Installation of 2-stop tubing for the Ismatec pump cassette. The length of the tubing should be adjusted to reach the flow-through reactors and influent source based on the position of the pump.

The tubing should be cut to the appropriate length to connect the influent source to the flow-through reactors. Autoclave the tubing prior to use and change it weekly during experiments to prevent biofouling and maintain tubing integrity due to the inevitable wear from the peristaltic pump. We recommend keeping the inlet tubes above the water column to prevent biofouling. We used heavy blunt end Luer-lok needles linked to tubing with a male tubing connector to weigh the inlet tubing down to the bottom of the influent bucket regardless of the water level. The 2-Stop tubing is designed to be installed in the Ismatec pump cassettes that hold the tube on the peristaltic rollers. A pump cassette with two-stop tubing installed is shown in Fig. 8.

**Fig. 9:**
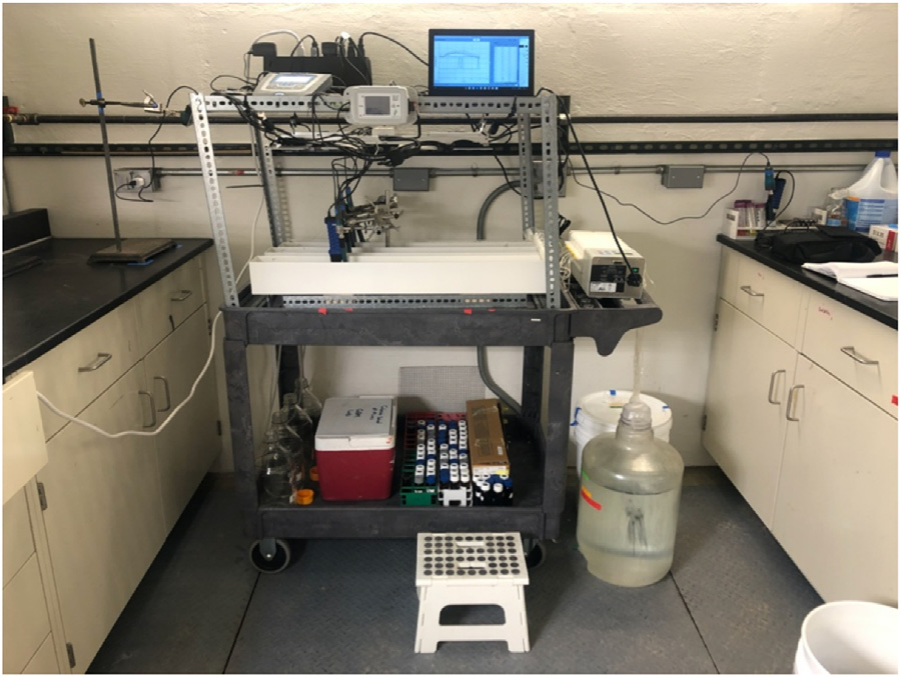
Assembled flow-through reactors fitted to a utility car with frame to support the lights and other electronics.

**Fig. 10:**
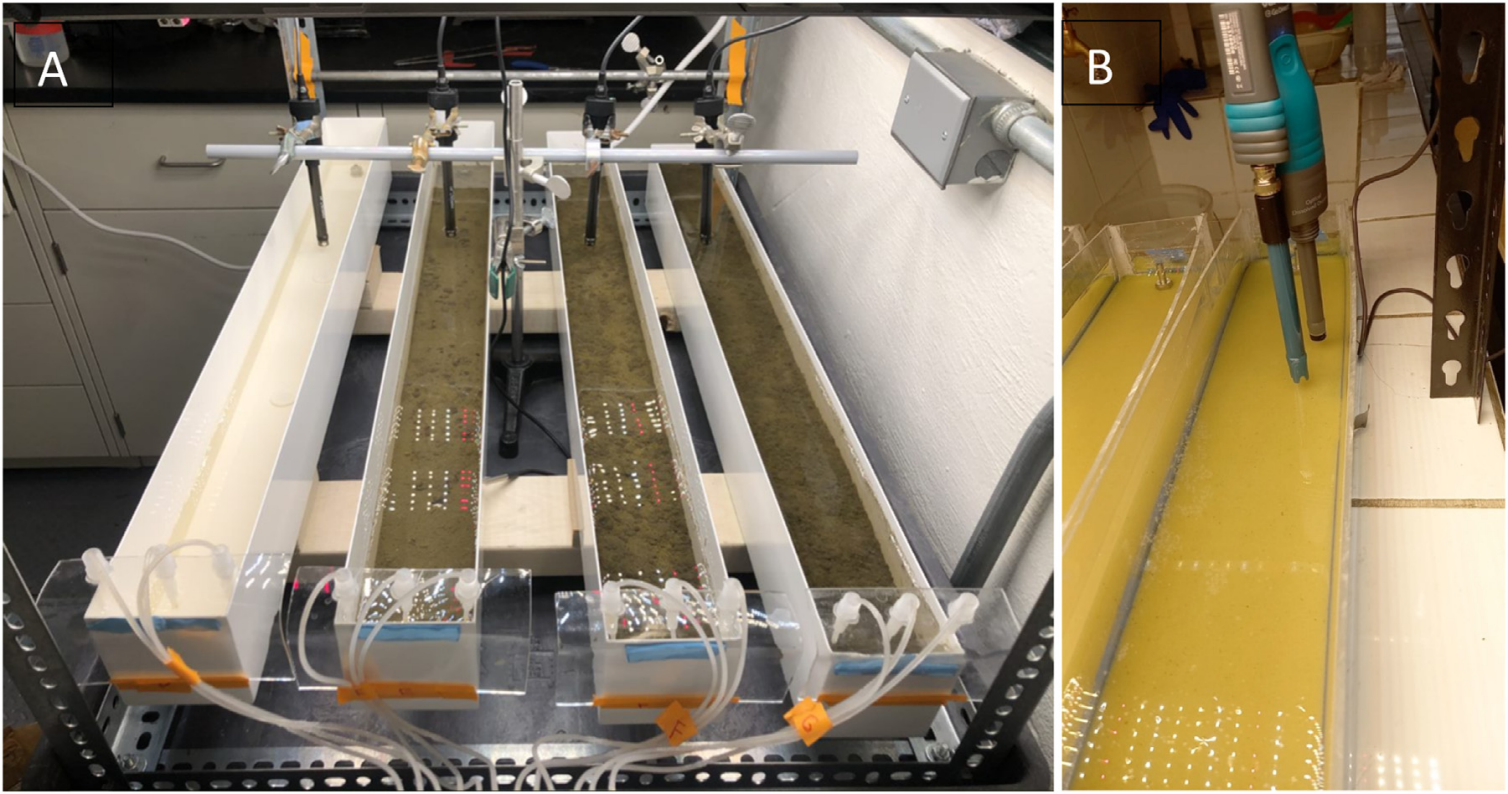
Flow-through reactors containing biomat and a biomat-free control. (A) Reactors are instrumented with probes held by a ring stand to continuously monitor pH, DO, and temperature. (B) Custom plexiglass flow-through reactors were created in a different laboratory to better view the biomat below the water line, demonstrating the adaptability of this design.

The pump can be placed on the handles of the cart and strapped with a utility cord or straps, if necessary. If a larger pump is used, it can be placed on top of the frame or under the cart. Make sure the pump is raised if it is placed under the cart in the event of influent or effluent overflow caused by loose tubing or clogs. A complete set of flow-through reactors containing DO, temperature and pH probes is shown in Figs. 9 and 10, respectively. Calibrate the pump using the bucket and stopwatch method by recording the weights of empty containers, pumping liquid into them for a set time, recording the end mass, and determining the flow rate. We recorded the flow rates in triplicate for three different pump settings to create a linear regression to predict the setting required for a desired flow rate.

We used 2 L media bottles or smaller sample vials to collect effluent for experiments, but the drain lines can be cut long enough to reach a floor drain or other waste container if effluent collection is unnecessary. The flow-through reactors were mounted on a wooden frame with furniture levelers. A plexiglass sheet was set on top of the steel frame to serve as a shelf for meters and cords. A ring stand with a horizontal bar and adjustable clamps was placed in the middle to hold the pH, DO, and conductivity probes.

### Establishment of field-derived photosynthetic microbial mats in the laboratory

(1) Obtain and process field samples. We manually collected approximately 6.5 L of a surficial portion (top several centimeters) benthic biomat from the field site using 1 L wide mouth Nalgene polypropylene bottles. The harvested biomat was stored and transported at 4 °C. This step will vary according to the system, but the goal is to obtain a representative sample that maintains viability.
(2) Thoroughly clean the flow-through reactors. We perform a dilute bleach soak, a water rinse, and 70% ethanol wipe down followed by air drying.
(3) Mark the interior of the flow-through reactors with 25 mm vertical increments using an indelible marker, or deploy rulers to monitor biomat depth.
(4) With clean, autoclaved utensils and a large mixing bowl, homogenize the biomat or sediment from the field sample and add to the flow-through reactors with a target depth of approximately 1 cm (this is potentially an experiment-specific variable).
(5) Add influent media to desired depth and let the benthic biomat settle for approximately 12 h with the grow lights off. The settling time limits the sediment from flowing out of the reactors when influent flow begins.
(6) Start the desired flow of influent representative of field waters, or a medium modified for experimental needs. We use a flow rate that creates a hydraulic residence time in the open water column of 12 – 48 h, depending on the experimental goals. Examples of flow rate and media calculations are included in the supplementary information.

While the depth of biomat and open water can be adjusted to experimental needs, we recommend adding at least 1 cm of biomat material to capture the diverse microbial consortia living both above and below the biomat oxic-anoxic interface, which is inferred to be within the surficial 1 cm of the biomat-water interface [13]. For the purposes of method validation with readily available data, biomat from the Prado Constructed Wetlands (Orange County, California) was used for seeding of the flow-through reactors. The depth of the water column was 3.5 cm and the depth of the biomat was 2 cm. The diel cycling of pH and DO is typically observed within 3 days of flow start-up, with steady-state diel cycling observed in 5–7 days.

Especially for experiments where the fate and transport of nutrients or trace inorganics are of interest, a parallel biomat-free reactor aids in hypothesis testing. We have observed nitrogen transformation in biomat-free systems, presumably due to adsorption to materials such as pump tubing, or microbial contamination within flow lines or influent containers (data not shown). Material compatibility can also be an issue, as we identified trace aluminum leaching from raw aluminum flow-through reactors that complex with trace metalloids in water (data not shown). Laboratory environmental variables such as humidity and elevation, and instrument accuracy and precision are easier to understand by including a biomat-free control reactor operated in parallel to the experimental ones.

## Influent growth media

We use a composition intended to replicate water from the Santa Ana River as sampled near Corona, CA (Table 3). A portion of the Santa Ana is diverted into the Prado Constructed Wetlands, which contain UPOW cells of different configurations as well as vegetated cells [11,13]. Synthetic river water was supplemented with trace metals and silica to maintain microbial growth and resident diatoms. This synthetic water recipe was based on cation and anion data from the Prado Constructed Wetlands field site [11]. The recipe was initially designed for batch experiments that lasted two weeks or less, where the biomat pore water would provide enough trace elements and nutrients to microorganisms when mixed with synthetic field water. Here, we improve the recipe by providing a steady supply of trace elements to maintain viability and activity for an extended period. A trace metals solution was used based on SL-8 in Atlas’ Handbook of Microbiological Media [14]. This information is intended to provide insight into the decisions made for the influent, but the influent can be tailored to the specific system and experimental conditions being studied. Using the design and materials described, triplicate flow-through reactors containing a total depth (free water plus biomat) of 4.8 cm, operated at a 24-h hydraulic retention time, require 8.4 l of media per day.

### Silica Stock

1. Measure 0.2260 g Na_2_SiO_3_•5H_2_O (sodium metasilicate pentahydrate) into 1 L of Milli-Q® water (1 mM final stock concentration).
2. Store in 1 L media bottle at room temperature.

### Metals stock

1. Measure the components in Table 2 into one liter of Milli-Q® quality water.

**Table 2:** Metals stock reagents.

**Compound**	**Mass (g)**	**Conc. (mM)**
disodium EDTA	5.20	15.37
FeCl_2_•4H_2_O	1.50	7.55
CoCl_2_•6H_2_O	0.19	1.46
MnCl_2_•4H_2_O	0.10	0.50
ZnCl_2_	0.07	0.51
H_3_BO_3_	0.06	1.0
NaMoO_4_•2H_2_O	0.04	0.15
CuCl_2_•2H_2_O	0.02	0.10
NiCl_2_•6H_2_O	0.02	0.19

2. Filter the stock from a 1 L volumetric flask into an autoclaved, dark (taped or foiled), 1 L media bottle with an autoclaved stir bar inside and store at 4 °C. Having a smaller (100–250 mL) autoclaved stock bottle with a stir bar for shorter term use/storage is recommended to minimize contamination in the main stock bottle.

Preparation of Media

1. When preparing a 5-gallon bucket for use with media, use a graduated cylinder to measure 20 L of tap water into the bucket. Mark the top of the water with tape or an indelible pen on the bucket as a fill line for future reference, then empty the bucket.
2. The bucket and its lid are cleaned by rinsing and scrubbing with diluted Alconox detergent, rinsing with tap water three times, rinsing with dilute bleach, and then rinsing with deionized (DI) water three times.
3. Prepare the media in a 5-gallon bucket. Fill both containers ∼½ to ¾ full with DI water.
4. Pipette 1 mL of Si stock into each container.
5. Pipette 20 mL of the metals stock into each container.
6. Mass and add the compounds in Table 3 into each 5-gallon bucket.

**Table 3:** Media reagents for growth media.

**Mass (g)**	**Conc. (mM)**	**Salt Compound**
2.46	0.5	MgSO_4_•7H_2_O
4.7	2.12	CaCl_2_
0.19	0.05	K_2_HPO_4_
6.05	3.6	NaHCO_3_
0.99	0.35	Na_2_SO_4_
0.49	0.33	KCl
1.28	1.1	NaCl
0.66	0.39	NaNO_3_
7. Top off to the 20 L fill line from Step 1 and mix the media with a sterile instrument.
8. We loosely seal the bucket with the lid so that the lid can be easily taken off for sampling and store the medium at 4 °C until used.
9. We typically have 3 media buckets in rotation: making a batch of media in 2 buckets at once, while the third bucket is actively providing media to the flow cells.

## Method validation

### Diel cycling

**Fig. 11:**
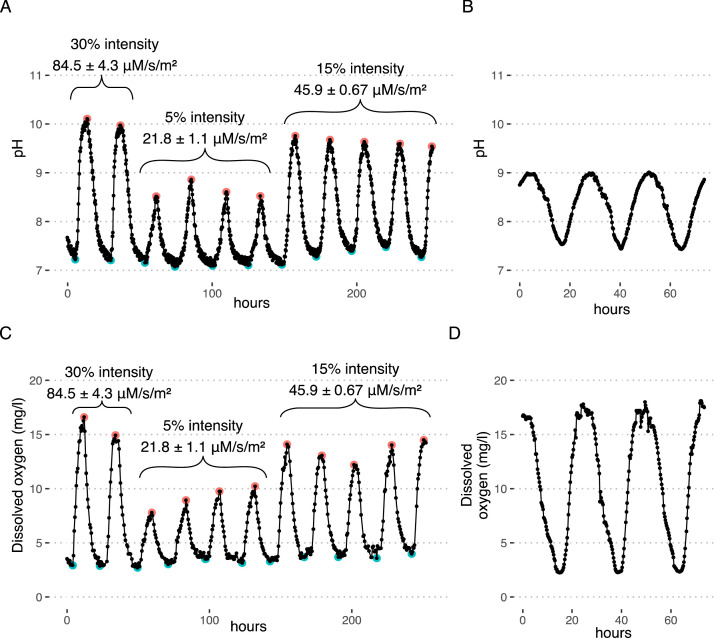
Tuning pH (panel A) and dissolved oxygen (panel C) in lab-scale flow-through reactors to approximate Prado Constructed Wetlands diel cycling (panels B, D) [15]. The blue points represent lights turning on and the red points represent lights turning off. The light intensity values represent the mean and standard deviation of three measurements along the length of the flow-through reactors.

Laboratory-scale UPOW wetland reactors represent a tunable system capable of modifying overlying water pH and dissolved oxygen based on light cycling and influent nutrient parameters. For method validation, we monitored the pH and dissolved oxygen of the water column above the biomat at 15 to 30-min intervals for multiple 24 h diel cycles (lights 12 on / 12 off). The light intensities varied at 5, 15 and 30% relative to their maximum light output, reported in PPFD at the water surface (Fig. 11 panels A & C). As a result, on average we were able to control the daily pH cycling between 7.2 - 10.2 (Δ 3), 7.4 - 9.6 (Δ 2.2), and 7.1 - 8.7 (Δ 1.6) for relative light intensities of 30, 15, and 5%, respectively. Similarly, dissolved oxygen fluctuated between 2.9 −16.1 (Δ 13.2), 3.7 - 15.5 (Δ 11.8), and 3.2 - 9.1 (Δ 5.9) mg/l, respectively. Maxima in both cases corresponded to the presence of light. These diel cycle results approximate field-relevant values seen within demonstration-scale UPOW wetlands situated within the Prado Constructed Wetlands facility in Corona, CA [11,15] (Fig. 11 panels B & D). In June 2018, pH experienced diel cycles between 7.4 - 9 (Δ 1.6), and dissolved oxygen between 2.3 −18.1 (Δ 15.9) mg/l. To date, controlling pH and DO cycles using naturally occurring inorganic buffers such as phosphate or bicarbonate has resulted in limited success, as it appears that natural buffering provided by the microbial mat exerts a stronger pressure. When relevant to experimental goals, further optimization of light intensity and diel cycle times and windows can improve how model lab-scale UPOW wetlands represent field-relevant processes.

## CRediT authorship contribution statement

**Gary Vanzin:** Conceptualization, Methodology, Writing – original draft, Validation, Formal analysis, Visualization. **Henry Peel:** Conceptualization, Methodology, Writing – original draft. **Weishi Wang:** Methodology, Validation, Writing – review & editing. **Lily Bosworth:** Methodology, Writing – review & editing. **Zhaoxun Yang:** Methodology, Writing – review & editing. **Michael A.P. Vega:** Methodology, Writing – review & editing. **Colin Root:** Conceptualization, Methodology, Writing – review & editing. **Adam Brady:** Conceptualization, Methodology, Writing – review & editing. **Giuliana Romero Mariscal:** Methodology, Validation, Writing – review & editing. **Armando Arenazas Rodríguez:** Methodology, Validation, Writing – review & editing. **Juana Ticona:** Methodology, Validation, Writing – review & editing. **Lino Morales Paredes:** Methodology, Validation, Writing – review & editing, Supervision, Funding acquisition. **Jonathan O. Sharp:** Methodology, Validation, Conceptualization, Writing – review & editing, Supervision, Funding acquisition.

## Declaration of Competing Interest

The authors declare that they have no known competing financial interests or personal relationships that could have appeared to influence the work reported in this paper.

## Data Availability

Data will be made available on request.

## Appendix A Material purchasing links

(1) Amazon.com: Modern Rectangle Planter Box - 32″ Metal Planter Ideal as a Long Succulent Planter | Rectangular Planter Box for Table or Window Sill Planters Indoor | Trough Planter for Indoor Window Planter | White : Patio, Lawn & Garden https://www.amazon.com/dp/B086RGJ3F6/?coliid=I27B51N5JLVQOX&colid=MYDL8VPC7N4J&ref_=lv_ov_lig_dp_it&th=1 (accessed 2022 −01 −30).
(2) Amazon.com: Lenofocus LF2000 LED Grow Light with Samsung LM301B Diodes Meanwell Driver, 3 × 3 4 × 4 4 × 2 FT Full Spectrum LED Board Grow Lights for Indoor Plants, Dimmable Plant Growing Lamp for Seeding Veg Flowering : Patio, Lawn & Garden https://www.amazon.com/Upgraded-Lenofocus-Dimmable-Hydroponics-Flowering/dp/B08C7GLJ8H/ref=sr_1_1?dchild=1&keywords=dimmable%2Bled%2Bgrow%2Blight%2B10v&s=lawn-garden&sr=1–1&th=1 (accessed 2022 −01 −30).
(3) FGI 0–10 V Universal Dimming Controller V2 https://forevergreenindoors.com/products/led-controller (accessed 2022 −01 −30).
(4) Amazon.com: WEN 73,004 500-Pound Capacity 36 by 24-Inch Extra Large Service Utility Cart : Everything Else https://www.amazon.com/WEN-73004-Capacity-Service-X-Large/dp/B0199R9WH6/ref=sr_1_6?dchild=1&keywords=lab+cart&qid=1631818975&sr=8–6 (accessed 2022 −01 −30).
(5) Stainless Steel Swagelok Tube Fitting, Bulkhead Union, 1/4 in. Tube OD | Bulkheads | Tube Fittings and Adapters | Fittings | Swagelok https://products.swagelok.com/en/c/straights/p/SS-400–61 (accessed 2022 −01 −30).
(6) Ferrule Set (1 Front Ferrule/1 Back Ferrule) | Spare Parts and Accessories | Tube Fittings and Adapters | Fittings | Swagelok https://products.swagelok.com/en/c/ferrule-sets/p/SS-400-SET (accessed 2022 −01 −30).
(7) Tubing | Tubing and Tube Accessories | Swagelok https://products.swagelok.com/tubing-tube-accessories/tubing/c/811?clp=true (accessed 2022 −01 −30).
(8) Ismatec - Tubing http://www.ismatec.com/int_e/tubing/2-stop/tygon_standard.htm (accessed 2022 −01 −30).
(9) Masterflex Transfer Tubing, Tygon® E-Lab (E-3603), 1/4″ ID x 5/16″ OD; 50 Ft from Cole-Parmer https://www.coleparmer.com/i/masterflex-transfer-tubing-tygon-e-lab-e-3603–1–4-id-x-5–16-od-50-ft/0640705 (accessed 2022 −01 −30).
(10) Masterflex Fitting Kit, Polypropylene, Straight/Reducer/Plug, Hosebarb, Small Fittings from Cole-Parmer https://www.coleparmer.com/i/masterflex-fitting-kit-polypropylene-straight-reducer-plug-hosebarb-small-fittings/4151403 (accessed 2022 −01 −30).
(11) Ismatec - IPC, IPC—N, tubing pump, peristaltic pump, multi-channel http://www.ismatec.com/int_e/pumps/t_ipc/ip_ipc.htm (accessed 2022 −01 −30).
(12) Everbilt 1/2 in. Black Neoprene Grommet 812,028 https://www.homedepot.com/p/Everbilt-1–2-in-Black-Neoprene-Grommet-812028/204276111 (accessed 2022 −02 −06).
(13) OATEY 14 oz. Plumber's Putty 311,662 https://www.homedepot.com/p/OATEY-14-oz-Plumber-s-Putty-311662/202312407 (accessed 2022 −02 −06).
(14) Amazon.com: Adjustable Feet Levelers, Heavy Duty Adjustable Legs for Furniture, Large Furniture Levelers, T Nut Levelers Table Adjustable Feet 3/8–16 Thread (4 Pack, Black) : Tools & Home Improvement. https://www.amazon.com/Adjustable-Levelers-Heavy-Furniture-Thread/dp/B09CYLYFJT/ref=asc_df_B09CYLYFJT/?tag=hyprod-20&linkCode=df0&hvadid=550276586843&hvpos=&hvnetw=g&hvrand=2806117855731823484&hvpone=&hvptwo=&hvqmt=&hvdev=c&hvdvcmdl=&hvlocint=&hvlocphy=1014524&hvtargid=pla-1435046291426&th=1 (accessed 2022–05–10).

## Appendix B Supplementary materials

Supplementary material *and/or* additional information Image, application 1
